# Epidemiology and outcome of sepsis in adult patients with *Streptococcus pneumoniae* infection in a Norwegian county 1993–2011: an observational study

**DOI:** 10.1186/s12879-016-1553-8

**Published:** 2016-05-23

**Authors:** Åsa Askim, Arne Mehl, Julie Paulsen, Andrew T. DeWan, Didrik F. Vestrheim, Bjørn Olav Åsvold, Jan Kristian Damås, Erik Solligård

**Affiliations:** Clinic of Anaesthesia and Intensive Care, St Olav University Hospital, Trondheim, Norway; Department of Circulation and Medical Imaging, Norwegian University of Science and Technology, Trondheim, Norway; Centre of Molecular Inflammation Research Department of Cancer Research and Molecular Medicine, Norwegian University of Science and Technology, Trondheim, Norway; Department of Medicine, Levanger Hospital, Nord-Trøndelag Health Trust, Levanger, Norway; Department of Public Health, Norwegian University of Science and Technology, Trondheim, Norway; Department of Infectious Diseases, St Olav University Hospital, Trondheim, Norway; Department of Endocrinology, St Olav University Hospital, Trondheim, Norway; Department of Chronic Disease Epidemiology, Yale School of Public Health, New Haven, CT USA; Norwegian Institute of Public Health, Oslo, Norway; Middle Norway Sepsis Research Center, Norwegian University of Science and Technology, Trondheim, Norway; Faculty of medicine, Department of Circulation and Medical Imaging, Po box 8905, N-7491 Trondheim, Norway

**Keywords:** *Streptococcus pneumoniae*, Bacteremia, Sepsis

## Abstract

**Background:**

Invasive pneumococcal disease (IPD) is responsible for significant mortality and morbidity worldwide. There are however few longitudinal studies on the changes in case fatality rate of IPD in recent years. We carried out a prospective observational study of patients with IPD in Nord Trøndelag county in Norway from 1993 to 2011 to study the clinical variables and disease outcome. The main outcome was all-cause mortality after 30 and 90 days.

**Methods:**

Patients with positive blood cultures were registered prospectively by the microbiology laboratory and clinical variables were registered retrospectively from patients’ hospital records. The severity of sepsis was assigned according to the 2001 International Sepsis Definition Conference criteria. The association between mortality and predictive factors was studied using a logistic regression model.

**Results:**

The total number of patients was 414 with mean age of 67 years and 53 % were male. Comorbidity was assessed by the Charlson Comorbidity Index (CCI). A CCI-score of 0 was registered in 144 patients (34.8 %), whereas 190 had a score of 1–2 (45.9 %) and 80 (19.3 %) had a score ≥3. 68.8 % of the patients received appropriate antibiotics within the first 6 h. The 30-day mortality risk increased by age and was 3-fold higher for patients aged ≥80 years (24.9, 95 % CI 16.4–33.4 %) compared to patients aged <70 (8.0, 95 % CI 3.5–12.4 %). 110 patients, (26.6 %) had severe sepsis and 37 (8.9 %) had septic shock. The 30 day all-cause mortality risk for those with sepsis without organ failure was 5.4 % (95 % CI 2.7–8.0 %), 20.2 % (95 % CI 13.5–27.4 %) for those with severe sepsis and 35.0 % (95 % CI 21.6–49.0 %) for those with septic shock. The mortality risk did not differ between the first and the second halves of the study period with a 30-day mortality risk of 13.5 % (95 % CI 7.9–19.2 %) for 1993–2002 versus 11.8 % (95 % CI 8.2–15.3 %) for 2003–2011.

**Conclusion:**

IPD carries a high mortality despite early and appropriate antibiotics in most cases. We found no substantial decrease in case fatality rate during the study period of 18 years. Older age and higher severity of disease were important risk factors for death in IPD.

**Electronic supplementary material:**

The online version of this article (doi:10.1186/s12879-016-1553-8) contains supplementary material, which is available to authorized users.

## Background

*Streptococcus pneumonia* colonizes the upper airway in approximately 10 % of adults and is responsible for the majority of episodes of community-acquired pneumonia [1, 2]. It may penetrate beyond epithelial cells and cause invasive disease and bacteremia [3]. Invasive pneumococcal disease (IPD) and sepsis are responsible for substantial morbidity and mortality worldwide with about 2 million deaths each year [4]. The case fatality rate of IPD and sepsis is 11–30 % in studies in Europe and the US [5]. The incidence of IPD is highest in the <2 and >65 year age groups and in the older population, chronic comorbid disease is a risk factor for acquiring IPD [2]. The 23-valent pneumococcal polysaccharide vaccine (PPV23) has been recommended to risk-groups and adults aged ≥65 years since 1996. The uptake is limited, and has been estimated to be about 15–30 % [6]. The 7-valent pneumococcal conjugate vaccine (PCV7) was introduced in the childhood immunization program in July 2006, and was replaced by the 13-valent vaccine (PCV13) in 2011. Since 2009, the uptake has been about 92 % for the complete immunization schedule [7].

The development of fatal pneumococcal sepsis depends on a complex interplay between the host and the microbe [8, 9]. Host factors such as diabetes, liver disease and COPD are associated with increased case fatality rate among immunocompetent patients. In the immunocompromised patients, HIV-infection, chronic renal failure and malignant disease have been associated with increased case fatality [10, 11]. Various guidelines have been developed to describe the optimal management of IPD and sepsis, such as the Surviving sepsis campaign [12] and guidelines for management of severe community acquired pneumonia [13]. These have had an impact on early recognition and treatment of septic patients, for instance by underscoring the importance of early antibiotic treatment [14]. However, important determinants of poor outcome of the disease remain to be identified to improve the management and treatment of IPD patients. We carried out a prospective observational study of risk factors for 30 and 90-day mortality among patients with IPD and sepsis in Nord-Trøndelag county, Norway from 1993 to 2011.

## Methods

### Setting and population

Nord-Trøndelag is a county in Central Norway with a population of approximately 134 000. It has both coastal and inland municipalities and consists of rural areas as well as small cities, but lack big cities. It is served by two community hospitals, Namsos Hospital and Levanger Hospital. The closest tertiary referral hospital is St Olav’s Hospital, Trondheim University Hospital. We included all patients ≥16 years of age with IPD confirmed by growth of *S.pneumoniae* in blood culture or cerebrospinal fluid at Levanger Hospital between 1993 and 2011, and at Namsos Hospital between 1999 and 2011. Residents of Nord-Trøndelag with IPD detected at St Olav’s Hospital between 1993 and 2011 were also included if they participated in a population survey (the HUNT-2 Survey) between 1995 and 1997 [15].

BACTEC 9240 (Becton Dickinson Diagnostic Instrument Systems, Sparks, MD) was used for blood culture testing until the beginning of 2010 and thereafter the Bactec FX [16]. Spinal fluid samples were cultured on chocolate plates incubated in an atmosphere of 5 % CO2 for 2–3 days. *S. pneumoniae* was identified by typical colony morphology, microscopic morphology, and optochin sensitivity. Antibiotic susceptibility testing and screening for penicillin resistance (oxacillin 1 μg disk) were performed by the disc-diffusion method (Oxoid, UK). Penicillin MIC determination (E test, AB Biodisk, Sweden) was performed in all pneumococcal isolates. Antimicrobial susceptibility was categorized according to breakpoints from EUCAST (www.eucast.org). Penicillin non-susceptibility was categorized using the break-point for meningitis cases (MIC >0.064 mg/l).

## Serotyping

*S. pneumoniae* isolates from cases of IPD were forwarded to the National Reference Laboratory for Pneumococci at the Norwegian Institute of Public Health. Isolates were confirmed as pneumococci, and serotyped by the Quellung reaction using serotype-specific antisera (Statens Seruminstitut, Copenhagen, Denmark) [17].

## Patient characteristics

All patients with positive blood cultures were prospectively registered and clinical information was extracted retrospectively from the patients’ hospital records. All data were collected using a standardized data retrieval form assessing patients’ characteristics, comorbid conditions, results of investigations and treatment. The data collection was carried out by trained research nurses and all registered data was secondarily assessed by an infectious disease consultant or the first author of the study. An episode of bloodstream infection was defined as the presence of one or more microorganism(s) in blood culture along with clinical evidence of infection. If a patient had more than one episode of *S. pneumoniae* bacteremia during the study period, only the first was included.

The setting of infection was classified as hospital-acquired (HA), healthcare- associated (HCA) or community-acquired (CA) as defined by Friedman et al. [18]. Patients who were hospitalized for two or more days during the 30 days prior to the infection were classified as having a HCA infection, in keeping with the definition used by Siegman-Igra et al. [19]. The number and severity of combined comorbid conditions were assessed according to the Charlson weighted Comorbidity Index (CCI) [20]. Case fatality rate was measured as all-cause mortality within day 30 and 90. By using the 11-digit unique identification number of all Norwegian citizens, electronic hospital records in Norway are updated with mortality data from the Norwegian population registry so that mortality data after discharge from hospital can be reliably assessed [21, 22].

## Severity of disease

Severity of disease (sepsis without organ failure, severe sepsis and septic shock) was determined according to the 2001 International Sepsis Conference definition [23]. We defined sepsis as documented bloodstream infection and two or more of the following: temperature ≥38.3 °C or <36.0 °C, heart rate >90 beats/min, respiratory rate >20/min or PaCo2 < 4.3 kPa or mechanical ventilation due to acute respiratory failure, glucose >7.7 mmol/l in the absence of diabetes, leucocytes >12 × 10^9^/l or <4 × 10^9^/l, elevated CRP or procalcitonin, acute hypotension (systolic BT <90 mmHg, MAP <70 mmHg or a fall of ≥40 mmHg), or need of fluid resuscitation (>20 ml/kg over 24 h). The specific criteria for organ dysfunction used were the same as those outlined in the 2001 International Sepsis Conference definition [23]. Any changes in disease severity were registered. The most severe deterioration of organ function was noted and used to define whether the patient had sepsis, severe sepsis or septic shock. For those who did not show any evidence of deterioration during the episode, the day of positive blood culture was registered as the day with the most severe affection. Severity of disease was also assessed by the Pitt bacteremia score [24].

## Focus of infection

Lower respiratory tract focus was diagnosed with clinical signs of respiratory infection and positive radiologic findings. Reported signs of infection along with focal growth of the same microbe as in the blood culture was taken as a confirmation of skin, soft tissue, joint or surgical infection. Meningitis was diagnosed with clinical signs and growth of the microbe in the cerebrospinal fluid. Upper respiratory tract infection was defined as otitis media or sinusitis. Otitis media was diagnosed as inflammation and accumulation of infected fluid of the middle ear and fever. Sinusitis was defined as inflammation of the sinuses, fever, pain in the face or headache and the presence of thick nasal mucus. An abscess was defined as a localized collection of pus in a tissue together with bacteremia. An unknown focus of infection was assigned when none of the criteria for ascertaining a focus were met.

## Initial antibiotic treatment

Appropriateness of initial antibiotic therapy (AIAT) was evaluated: initial therapy had to be given (1) intravenously in correct doses and timely after the blood culture specimen was obtained (<6 h or >6 h), (2) with a regimen that was active in vitro against the microbe(s) isolated from blood culture(s).

## Statistical analysis

Data was analyzed using SPSS for Windows (Version 21, Armonk, NY: IBM Corp) and STATA version 13 (Stata Corp LP, College Station, Texas). The associations between clinical characteristics and 30 and 90-day mortality were investigated using logistic regression analysis where odds ratios (ORs) with 95 % confidence intervals (CI) were estimated. In addition, we studied the association between clinical characteristics and severe sepsis/septic shock as an outcome, in order to identify groups of patients particularly vulnerable to develop severe disease and need a higher level of follow-up and care. All associations were estimated both unadjusted and adjusted for potential confounders, chosen based on prior knowledge of factors that may influence both the exposure variable under interest and the risk of the outcome (death or sepsis severity). All adjusted analyses were adjusted for sex and age group (≤70, 70–79 and ≥80 years). The association of the setting of infection, focus, severity, and time period were additionally adjusted for comorbid conditions using three categories of the CCI (0, 1–2 and ≥3). Adjusted 30- and 90-day mortality risks and risk of severe sepsis/shock were estimated from the logistic regression model by using the margins post-estimation command in Stata. For ordinal variables, we tested for linear trend across categories by using the categories as a continuous variable in the logistic regression model.

## Results

We identified 414 patients with IPD during the study period. All met the sepsis criteria. The mean age was 67 years and 52.9 % were male. In 78.1 % of the patients, the infection was community-acquired, 17.6 % of infections were healthcare-associated and 4.3 % were hospital-acquired. The all-cause mortality rate was 12.3 % at 30 days and 16.7 % at 90 days (Table 1). A CCI-score of 0 was registered in 144 patients (34.8 %), whereas 1–2 in 190 (45.9 %) and ≥3 in 80 (19.3 %) of the patients (Table 1).

**Table 1:** Patient and infection characteristics

Characteristic	N (%)
Patients included	414 (100)
All-cause mortality	
30-days	51 (12.3)
90-days	69 (16.7)
Sex	
Female	195 (47.1)
Male	219 (52.9)
Acquisition	
Community-acquired	323 (78.1)
Healthcare-associated	73 (17.6)
Hospital-acquired	18 (4.3)
Age category	
< 50	69 (16.7)
50–69	131 (31.6)
70–79	104 (25.1)
≥ 80	110 (26.6)
Comorbid conditions	
Malignancy	90 (21.7)
Renal failure	24 (5.8)
Diabetes mellitus	51 (12.3)
Hypertension	98 (23.7)
Coronary heart disease	84 (20.3)
Heart failure	40 (9.7)
Chronic pulmonary disease	96 (23.2)
Cerebral ischemic disease	40 (9.7)
Charlson comorbidity index (CCI)	
0	144 (34.8)
1–2	190 (45.9)
≥ 3	80 (19.3)
Severity of sepsis	
Sepsis without organ failure	267 (64.5)
Severe sepsis	110 (26.6)
Septic shock	37 (8.9)
Pitt bacteremia score	
0	126 (30.4)
1	131 (31.6)
2	72 (17.4)
≥ 3	85 (20.6)
Focus of infection	N (%)
Abdomen	4 (1.0)
Lower respiratory tract	344 (83.0)
Upper respiratory tract	11 (2.7)
Skin/soft tissue/abscess/myositis	13 (3.1)
Meningitis	26 (6.3)
Unknown	16 (3.9)
Time period	
1993–2002	134 (32.4)
2003–2011	280 (67.6)
Management	
Treatment in Intensive Care Unit(ICU)	136 (32.9)
Treatment with vasopressors	40 (9.6)
Ventilator treatment	16 (3.9)
Penicillin-sensitive *Streptococcus pneumoniae*	397 (97.8)^a^

^a^We had information about 406 isolates of *S.pneumoniae*

The 30-day mortality risk increased by age (p for trend <0.001). The 30-day mortality risk was 3 fold higher for patients aged 80 years or older (24.9, 95 % CI 16.4–33.4 %) compared to patients aged under 70, (8.0, 95 % CI 3.5–12.4 %) (Table 2). A positive association with age was also seen for the 90-day mortality risk (Additional file 1: Table S1).

**Table 2:** 30-day all-cause mortality in relation to patient characteristics prior to infection

Characteristic	No. of deaths within 30 days	30-day mortality within category (%)	Age- and sex-adjusted				
			Odds ratio	95 % CI	p	Mortality risk (%)	95 % CI
Age (years)							
< 70	12	6.0	1	Reference		8.0	3.5–12.4
70–79	15	14.4	2.72	1.21–6.07	0.02	14.0	7.4–20.6
≥ 80	24	21.8	5.57	2.63–11.78	<0.001	24.9	16.4–33.4
p for trend					<0.001		
Sex							
Male	33	15.1	1	Reference		15.4	10.8–20.1
Female	18	9.2	0.51	0.27–0.97	0.040	8.9	5.0–12.8
Charlson Comorbidity Index (CCI)							
0	12	8.4	1	Reference		12.9	6.3–19.5
1–2	20	10.5	0.67	0.30–1.51	0.34	9.3	5.5–13.1
≥ 3	19	23.8	1.54	0.66–3.60	0.32	18.1	10.8–25.4
p for trend					0.11		
Comorbidities ^a^							
Malignant disease	18	20.0	1.61	0.82–3.13	0.15	16.1	9.2–23.0
Renal failure	6	25.0	2.25	0.82–6.21	0.11	21.7	7.0–36.4
Diabetes mellitus	9	17.6	1.44	0.64–3.28	0.43	15.7	6.1–24.9
Hypertension	22	22.4	2.35	1.22–4.54	0.01	19.3	12.0–26.6
Coronary heart disease	16	19.0	1.05	0.52–2.11	0.75	12.6	6.7–18.6
Heart failure	6	15.0	0.62	0.23–1.62	0.35	8.7	1.9–15.4
Chronic pulmonary disease	10	10.4	0.73	0.34–1.55	0.49	10.0	4.3–15.8
Cerebral ischemic disease	7	17.5	1.03	0.40–2.50	0.93	12.4	6.7–18.6

^a^Those not having the condition were used as reference category

Females had lower 30-day mortality risk than males 8.9 % vs 15.4 % (OR 0.51 95 % CI 0.27–0.97) (Table 2). This association was nearly unchanged but less precise after adjusting for comorbid disease (OR 0.55 95 % CI 0.29–1.05) and after adjusting for severity of sepsis on admission, time to antibiotics and place of acquisition (OR 0.54 95 % CI 0.26–1.10). A similar gender association was also seen for 90-day mortality risk. (Additional file 1: Table S1).

The initial antibiotic treatment was mainly penicillin, which is the standard empiric treatment for community-acquired pneumonia in Norway or penicillin in combination with aminoglycoside, which is the standard empiric treatment for severe community-acquired pneumonia or sepsis of unknown origin period. Overall, 85.5 % received antibiotics according to national guidelines [25, 26], 97.8 % of the pneumococcal isolates were sensitive to penicillin. Of the 406 isolates, 397 were sensitive to penicillin, seven were intermediate and two were resistant. We did not have information about eight samples. Within 6 h, 68.8% of the patients received antibiotic treatment, 18.6 % received treatment after 6 h and data on timing of treatment were lacking for 12.6 % of the patients. Patients who received antibiotics before 6 h had a 30-day mortality risk of 11.9 % (95 % CI 8.5–15.3 %), which was similar to patients who received antibiotics after 6 h (13.2, 95 % CI 8.1–18.4 %) (Table 3).

**Table 3:** 30 day all-cause mortality in relation to disease acquisition, severity, focus and time period

Characteristic	No. of deaths within 30 days	30-day mortality within category (%)	Age-, sex- and comorbidity-adjusted^a^				
			Odds ratio	95 % CI	p	Mortality risk (%)	95 % CI
Place of acquisition							
Community acquired	34	10.6	1	Reference		10.9	7.6–14.3
Health-care associated	12	17.0	1.58	0.65–3.20	0.35	14.7	6.9–22.4
Hospital acquired	5	27.8	3.13	0.92–10.43	0.07	25.2	6.7–43.9
Severity							
Sepsis without organ failure	14	5.2	1	Reference		5.4	2.7–8.02
Severe sepsis	23	21.0	5.19	2.44–11.10	<0.001	20.2	13.5–27.4
Septic shock	14	37.8	13.58	5.32–34.60	<0.001	35.0	21.6–49.0
p for trend					<0.001		
Pitt bacteremia score							
0	12	9.5		Reference		9.4	4.5–14.3
1	7	5.4	0.47	0.17–1.28	0.14	4.9	1.4–8.3
2	8	11.1	1.45	0.53–3.94	0.47	12.7	4.9–20.5
≥3	24	28.2	5.10	2.12–11.65	<0.001	29.8	20.7–39.0
p for trend					<0.001		
Time period							
1993–2002	17	12.7	1	Reference		13.5	7.9–19.2
2003–2011	34	12.1	0.77	0.43–1.61	0.59	11.8	8.2–15.3
Time to antibiotics^b^							
<6 hours	33	14.1	1	Reference		11.9	8.5–15.3
>6 hours	18	11.5	1.17	0.58–2.34	0.65	13.2	8.1–18.4

^a^Adjusted for Charlson Comorbidity Index: 0, 1–2 and ≥3 ^b^Also adjusted for severity

With regard to severity of disease, 64.5 % of the patients had sepsis without organ failure, 26.6 % had severe sepsis and 8.9 % had septic shock. In patients with sepsis with no organ failure the 30- day mortality risk was 5.4 % (95 % CI 2.7–8.0 %), the 30-day mortality risk was almost 4-fold higher for patients with severe sepsis (20.2, 95 % CI 13.5–27.4 %) and more than 6-fold higher for patients with septic shock (35.0, 95 % CI 21.6–49.0 %) (Table 3). A similar association was seen for 90-day mortality risk (Additional file 1: Table S2)*.*

An increase in mortality risk according to severity was also observed for the Pitt bacteremia score, especially for patients with Pitt bacteremia score ≥3 (Table 3 and Additional file 1: Table S2).

There was no convincing association between CCI-score and 30-day mortality but patients with a CCI-score ≥3 had a twofold higher 90-day mortality risk (27.2, 95 % CI 18.8–35.7 %) compared to patients with a CCI score of 0 (12.9, 95 % CI 6.3–19.5 %) (Additional file 1: Table S1). With regard to individual comorbidities, hypertension was associated with increased 30-day mortality risk (OR 2.35, 95 % CI 1.22–4.54) (Table 2). A similar result was observed for 90-day mortality (Additional file 1: Table S1).

This study was carried out over a period of more than 18 years. The age, sex and comorbidity adjusted 30- day mortality risk did not differ between the first (1993–2002) and second (2003–2011) periods of the study (13.5, 95 % CI 7.9–19.2 %) vs (11.8, 95 % CI 8.2–15.3 %) (Table 3). The 90-day mortality risk tended to be higher in the first than the second period (20.6, 95 % CI 14.2–27.0 %) vs (14.9, 95 % CI 11.0–18.7 %) (Additional file 1: Table S2).

Patients with heart failure had the highest risk of acquiring severe sepsis or septic shock (58.1, 95 % CI 33.4–73.5 %) and patients with renal failure had a risk of 53.5 % (95 % CI 33.4–73.5 %). The risk of acquiring severe sepsis or septic shock was lower in the second half of the study with an adjusted risk of 29.9 % (95 % CI 24.5- 35.2 %) versus 47.3 % (95 % CI 38.9–55.7 %) (Table 4).

**Table 4:** Proportion of severe sepsis/septic shock according to prior patient characteristics and infection-related characteristics

Characteristic	No with severe sepsis or septic shock	Severe sepsis or septic shock in category (%)	Age-, sex- and comorbidity^a^-adjusted				
			Odds ratio	95 % CI	p	Risk of severe sepsis or septic shock (%)	95 % CI
Age (years)							
<70	73	36.5	1	Reference		35.0	28.7–42.0
70–79	33	31.7	0.81	0.48–1.37	0.44	30.8	22.0–39.8
≥80	41	37.2	1.24	0.75–2.10	0.39	40.7	30.8–50.5
p for trend					0.49		
Sex							
Male	82	37.4	1	Reference		37.6	31.2–44.0
Female	65	33.3	0.88	0.57–1.34	0.54	33.1	26.5–39.8
Charlson ComorbidityIndex (CCI)							
0	46	31.9	1	Reference		33.0	24.7–41.2
1–2	70	36.8	1.10	0.64–1.91	0.73	36.5	29.5–43.3
≥3	31	38.7	1.29	0.72–2.29	0.39	37.7	26.8–48.5
p for trend					0.37		
Comorbidities^a^							
Malignant disease	33	36.7	0.98	0.59–1.61	0.94	35.1	25.2–45.1
Renal failure	16	66.7	2.20	0.95–5.11	0.07	53.5	33.4–73.5
Diabetes mellitus	14	27,4	0.62	0.32–1.21	0.16	26..7	14.6–38.7
Hypertension	38	38.7	1.17	0.71–1.91	0.54	38.2	28.2–48.1
Coronary heart disease	37	30.3	0.66	0.38–1.15	0.14	28.3	18.4–38.2
Heart failure	23	57.5	2.82	1.39–5.74	0.004	58.1	42.0–74.1
Chronic pulmonary disease	39	40.6	1.34	0.83–2.15	0.23	40.7	30.8–50.5
Cerebral ischemic disease	12	30.0	0.69	0.33–1.43	0.32	28.3	14.4–42.2
Place of acquisition							
Community acquired	112	34.7	1	Reference		34.6	29.6–40.0
Health-care associated	25	34.2	0.95	0.54–1.68	0.87	33.6	23.0–45.7
Hospital acquired	10	55.6	2.37	0.88–6.39	0.09	55.7	32.5–76.6
Time periode							
1993–2002	63	47.0	1	Reference		47.3	38.9–55.7
2003–2011	84	30.0	0.47	0.30–0.72	0.01	29.9	24.5–35.2

^a^The association of age, sex, CCI and individual underlying comorbidities with the risk of severe sepsis or septic shock are not adjusted for comorbidity

Serotype-information was available in 373 patients (90 %). Before introduction of the 7-valent pneumococcal conjugate vaccine (PCV 7) in the childhood immunization in 2006, the most frequent serotypes were 4, 14, 1, 9 and 3. In the period 2007 to 2011 the most frequent serotypes were 7 F, 22 F, 4, 3 and 19A, of which only serotype 4 is included in PCV 7 (Additional file 2: Figure S1–3).

## Discussion

In our study, there were strong positive associations of age and disease severity with case fatality rate in patients with IPD and sepsis. We did not observe a reduction in mortality risk over the time of our study.

IPD is a severe disease that predominantly occurs in elderly people with comorbid disease [27]. The mortality risk was higher in patients >80 years of age than in younger patients. First, the clinical presentation of sepsis in elderly patients may differ from younger patients. Unspecific symptoms, like abdominal pain, confusion or specific organ symptoms like dyspnea, can delay the sepsis diagnosis and initiation of therapy [28]. Indeed a recent Norwegian study found that antimicrobial therapy was less in compliance with current recommendations and more delayed in elderly patients with sepsis [29]. Second, ageing of the immune system and impaired effect of vaccine also contribute to the mortality risk [27]. Also, elderly patients may not be admitted to the intensive care unit due to high comorbidity burden or an end of life care situation.

We assessed whether individual prior comorbid conditions influenced the risk of acquiring severe sepsis or septic shock and found this risk was highest for patients with heart failure and renal failure. This is probably because of reduced residual capacity to meet the hemodynamic burden associated with severe sepsis [27, 30]. Interestingly, we found that females had lower mortality risk than males for both 30-day and 90-day mortality. Males had a higher comorbidity burden than females, but adjusting for comorbid disease did not change the risk estimate, neither did adjusting for severity on admission, place of acquisition or time for antibiotic treatment. Differences in alcohol and smoking habits may have influenced the mortality risk but we could not examine that possibility [31, 32].

A high proportion of the patients in our study received antibiotics according to guidelines and within the first 6 h after admission in the hospital or in the hospital within 6 h after suspected invasive infection and sepsis. There was no significant reduction in mortality when comparing whether the patients received antibiotics within 6 h or after 6 h. Confounding by indication is a complicating factor when assessing the effect of antibiotic treatment and outcome. It is possible that those with more severe illness received antibiotic treatment earlier. We adjusted for age, sex, comorbid disease burden and severity but there may still be unadjusted differences between the groups. In addition, it is likely that there were variations in time from how long the patients had suffered from the infection prior to admission since almost 80 % of the patients had community- acquired infection.

As expected, we saw a change in the serotype pattern before and after 2006. The three most frequent serotypes after 2006 were 7 F, 22 F, and 3. A metaanalysis from 2010 found that serotypes 3,6A, 6B, 9 N and 19 F were associated with increased risk of death and these had high carrier prevalence, low invasiveness and were encapsulated in vitro [33]. We did not have the statistical power to examine differences in mortality between different serotypes. Following the introduction of PCV7 in the childhood immunization program in 2006 the incidence of IPD declined in all age groups as a result of indirect effect [17]. This decline continued after the switch to PCV13 in 2011 [7]. PCV13 has been licensed for prevention of IPD in all age-groups, and in 2013 the Norwegian Institute of Public Health updated the recommendations for use of pneumococcal vaccines to elderly and risk-groups with a recommendation to use PCV13 in addition to PPV23 for persons with a very high risk for IPD [34]. However, as the impact of PCV13 in the childhood immunization program continues to reduce circulation of PCV13 serotypes, the preventive potential of PCV13 given to adults and risk-groups is reduced over time [6].

We did not demonstrate any reduced mortality risk over the time of our study, but the risk of acquiring severe sepsis was significantly decreased in the later period. This may be for several reasons such as introduction of the pneumococcal vaccine in the childhood vaccination program which has shown to influence other age groups in the society [1] and improved patient management as part of a continuing awareness of severe infections and sepsis [35].

All patients had verified bacteremia and the blood culture were prospectively registered by the microbiology laboratory, which ensures a broad inclusion of all affected patients. Sepsis was diagnosed in IPD-patients independent of primary focus, microbiological etiology and host factors. This study has, however, some limitations. The clinical data were collected from hospital records at a later date which is inferior to prospective clinical assessment but detailed data were available on the majority of the patients. It is a strength of our study that all IPD cases were confirmed by growth in blood culture or, in a few cases, in cerebrospinal fluid. However, it is also possible to suffer from IPD without having verified bacteremia or growth in the cerebrospinal fluid, for example, if antibiotic treatment was given prior to blood sampling, and we could not identify and include such patients. The study was carried out only in one region in Norway and may not be generalizable, particularly outside Scandinavia. Regrettably, we did not have information on vaccine status, which would have been an interesting aspect to assess in this population.

## Conclusions

In conclusion, we found that older age and higher severity of disease were important risk factors for death among patients with IPD and sepsis. We found no substantial decrease in case fatality rate during the study period. The study contributes to the understanding of IPD and sepsis in Norway.

## Abbreviations

AIAT, appropriateness of initial antibiotic therapy; CA, community-acquired CCI Charlson Comorbidity Index; HA, hospital-acquired; HCA, Healthcare-associated; IPD, invasive pneumococcal disease; MIC, minimum inhibitory concentration; PCV, pneumococcal conjugate vaccine

## Additional files

Additional file 1: Table S1. 90-day mortality in relation to patient characteristics prior to infection. Table S1a. 90-day mortality in relation to gender. **Table S2.** 90-day mortality in relation to disease acquisition, severity, focus and time period. (DOCX 22 kb) Additional file 2: Figure S1. Serotypes 1993–2011. **Figure S2.** Serotypes 1993–2006. **Figure S3.** Serotypes 2007–2011. (DOC 106 kb)
