# Supplementary material for: Epidemiology and outcome of sepsis in adult patients with Streptococcus pneumoniae infection in a Norwegian county 1993–2011: an observational study
Source: BMC Infect Dis. 2016 May 23;16:223. doi: 10.1186/s12879-016-1553-8 (PMC4877975; doi:10.1186/s12879-016-1553-8)
Supplement: Additional file 1: Table S1. — 90-day mortality in relation to patient characteristics prior to infection. Table S1a. 90-day mortality in relation to gender. Table S2. 90-day mortality in relation to disease acquisition, severity, focus and time period. (DOCX 22 kb) [file 12879_2016_1553_MOESM1_ESM.docx]

**Additional file1**

**Table S1: 90-day all-cause mortality in relation to patient characteristics prior to infection**

| Characteristic | No. of deaths within 90 days | 90-day mortalitywithin category (%) | Age- and sex-adjusted | | | | |
| --- | --- | --- | --- | --- | --- | --- | --- |
|  |  |  | Odds ratio | 95% CI | p | Mortality risk (%) | 95% CI |
|  |  |  |  |  |  |  |  |
| **Age (years**) |  |  |  |  |  |  |  |
| < 70 | 15 | 7.5 | 1 | Reference |  | 10.1 | 5.1-15.1 |
| 70-79 | 19 | 18.3 | 3.45 | 1.70-7.01 | 0.005 | 20.7 | 13.0-28.4 |
| ≥80 | 35 | 31.8 | 4.00 | 3.32-12.95 | <0.001 | 33.0 | 23.7-42.2 |
| p for trend |  |  |  |  | <0.001 |  |  |
| **Sex** |  |  |  |  |  |  |  |
| Male | 43 | 19.6 |  | Reference |  | 20.1 | 15.0-25.1 |
| Female | 26 | 13.3 | 0.56 | 0.32-0.98 | 0.053 | 13.0 | 8.4-17.5 |
| **Charlson Comorbidity index (CCI)** |  |  |  |  |  |  |  |
| 0 | 12 | 8.3 | 1 | Reference |  | 12.9 | 6.3-19.5 |
| 1-2 | 29 | 15.3 | 1.27 | 0.60-2.70 | 0.52 | 13.4 | 9.0-17.9 |
| ≥3 | 28 | 35.0 | 3.44 | 1.55-7.65 | <0.002 | 27.2 | 18.8-35.7 |
| **Comorbidities*** |  |  |  |  |  |  |  |
| Malignant disease | 28 | 31.1 | 2.39 | 1.33-4.33 | 0.004 | 25.8 | 17.6-34.0 |
| Renal failure | 7 | 29.2 | 1.92 | 0.72-5.02 | 0.20 | 25.2 | 10.0-40.4 |
| Diabetes mellitus | 10 | 19.6 | 0.99 | 0.45-2.17 | 0.90 | 17.2 | 7.9-26.5 |
| Hypertension | 27 | 27.5 | 1.88 | 1.04-3.43 | 0.04 | 28.0 | 15.3-30.3 |
| Coronary heart disease | 19 | 22.6 | 0.82 | 0.40-1.49 | 0.45 | 14.5 | 8.4-20.7 |
| Heart failure | 12 | 30.0 | 1.15 | 0.52-2.47 | 0.75 | 18.0 | 8.6-27.5 |
| Chronic pulmonary disease | 12 | 12.5 | 0.58 | 0.29-1.17 | 0.13 | 11.9 | 5.8-18.1 |
| Cerebral ischemic disease | 9 | 22.5 | 0.91 | 0.40-2.08 | 0.82 | 15.6 | 6.4-24.9 |
|  |  |  |  |  |  |  |  |

*****Those not having the condition were used as reference category

**Table S1a 90-day all-cause mortality in relation to gender**

|  |  | | | **Age and comorbidity adjusted** | | | | **Extended adjustment**** | | |  |  |
| --- | --- | --- | --- | --- | --- | --- | --- | --- | --- | --- | --- | --- |
|  | **Sex** | No of deaths within 90 days | 90-day mortality within category (%) | Odds ratio | 95 %CI | p |  | Odds ratio | 95%CI | p |  | **95% CI** |
|  | Male | 43 | 19.6 | 1 | Reference |  |  | 1 | Reference |  |  | .4-24.1 |
|  | Female | 26 | 13.3 | 0.62 | 0.35-1.11 | 0.11 |  | 0.66 | 0.35-1.26 | 0.21 |  | 9.1-18.4 |
|  | | | | | | | | | | |  |  |

**Additionally adjusted for place of acquisition, time to antibiotics and severity

**Table S2: 90-day all-cause mortality in relation to disease acquisition, severity, focus and time period**

| Characteristic | No. of deaths within 90 days | 90-day mortality within category (%) | Age-, sex- and comorbidity-adjusted * | | | | | |
| --- | --- | --- | --- | --- | --- | --- | --- | --- |
|  |  |  | Odds ratio | 95% CI | p | Mortality risk (%) | | 95% CI |
| **Place of acquisition** |  |  |  |  |  |  | |  |
| Community acquired | 43 | 13.5 | 1 | Reference |  | 14.3 | | 10.6-18.0 |
| Health-care associated | 19 | 26.0 | 1.97 | 0.99-3.91 | 0.05 | 21.6 | | 12.8-30.3 |
| Hospital acquired | 7 | 38.9 | 3.93 | 0.68-2.39 | 0.02 | 34.3 | | 15.0-53.6 |
| **Severity** |  |  |  |  |  |  | |  |
| Sepsis without organ failure | 22 | 8.0 | 1 | Reference |  | 8.4 | | 5.2-11.6 |
| Severe sepsis | 30 | 27.2 | 4.92 | 2.51-9.63 | <0.001 | 26.4 | | 19.2-33.7 |
| Septic shock | 17 | 46.0 | 13.25 | 5.38-32.62 | <0.001 | 42.3 | | 28.8-55.9 |
| p for trend |  |  |  |  | 0.37 |  | |  |
| **Pitt bacteremia score** |  |  |  |  |  |  | |  |
| 0 | 15 | 11.9 | 1 | Reference |  | 11.7 | | 6.5-17.0 |
| 1 | 13 | 9.9 | 0.74 | 0.32-1.71 | 0.49 | 9.2 | | 4.6-13.7 |
| 2 | 9 | 12.5 | 1.35 | 0.52-3.52 | 0.53 | 14.6 | | 6.5-22.7 |
| ≥3 | 32 | 37.7 | 6.70 | 3.00-14.94 | <0.001 | 39.4 | | 30.1-48.8 |
| p for trend |  |  |  |  | <0.001 |  | |  |
| **Time period** |  |  |  |  |  |  | |  |
| 1993-2002 | 26 | 19.4 | 1 | Reference |  | 20.6 | | 14.2-27.0 |
| 2003-2011 | 43 | 15.4 | 0.63 | 0.35-1.13 | 0.12 | 14.9 | | 11.0-18.7 |
| **Time to antibiotics**** |  |  |  |  |  |  | |  |
| < 6hours | 46 | 16.0 | 1 | Reference |  | 16.6 | | 12.9-20.4 |
| >6 hours | 23 | 18.1 | 1.01 | 0.53-1.94 | 0.97 | | 16.7 | 11.2-22.3 |

*Adjusted for Charlson Comorbidity Index: 0, 1-2 and ≥3 **Also adjusted for severity
