# Supplementary material for: Epidemiology and outcome of sepsis in adult patients with Streptococcus pneumoniae infection in a Norwegian county 1993–2011: an observational study
Source: BMC Infect Dis. 2016 May 23;16:223. doi: 10.1186/s12879-016-1553-8 (PMC4877975; doi:10.1186/s12879-016-1553-8)

BMC Infectious Diseases, 2016 Åsa Askim

Additional file 2

Figure 1


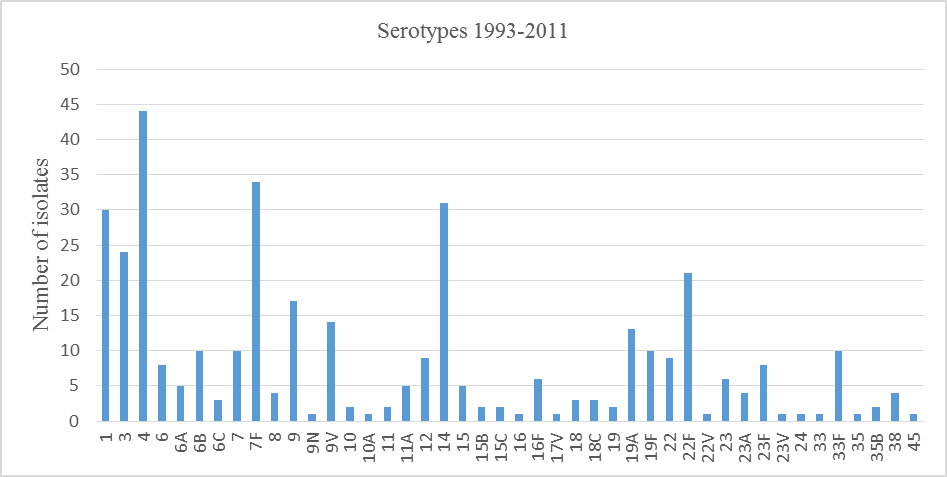


BMC Infectious Diseases, 2016 Åsa Askim

Additional file 2

Figure 2


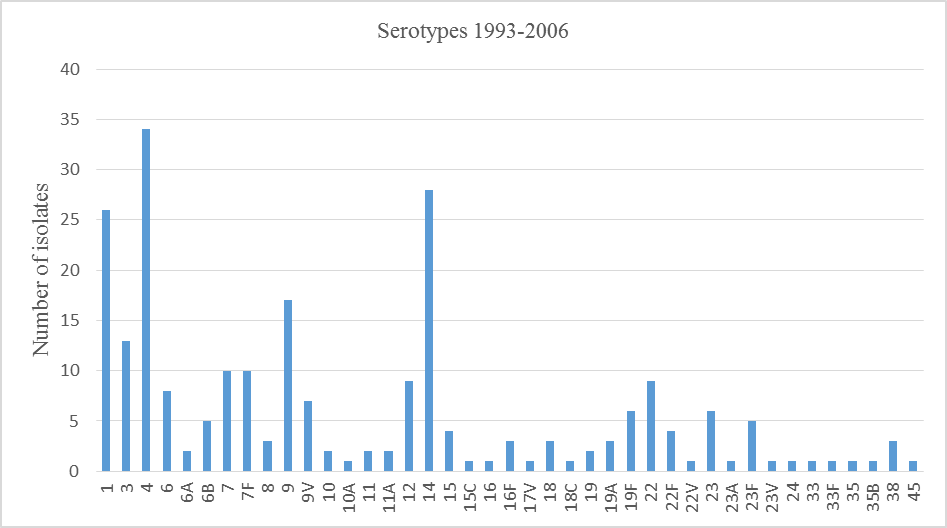


BMC Infectious Diseases, 2016 Åsa Askim

Additional file 2

Figure 3


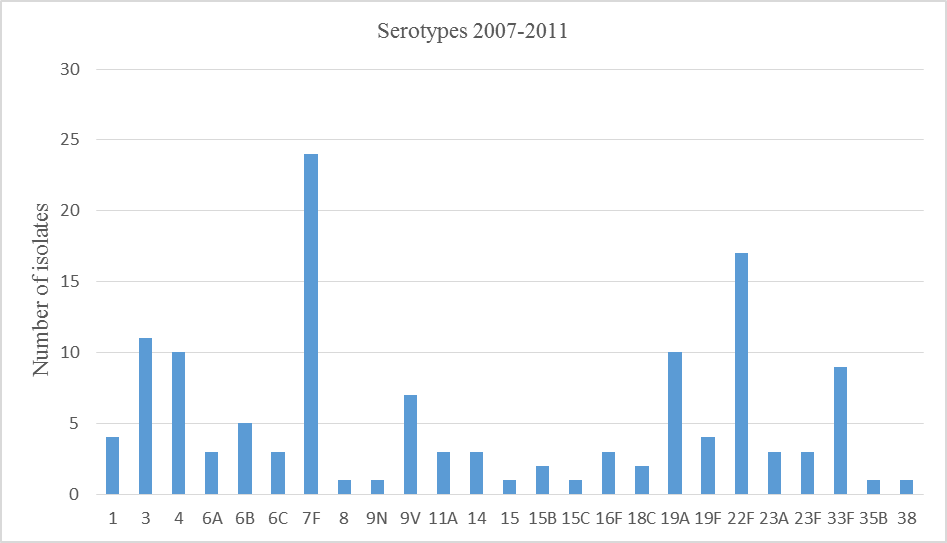

Supplement: Additional file 2: Figure S1. — Serotypes 1993–2011. Figure S2. Serotypes 1993–2006. Figure S3. Serotypes 2007–2011. (DOC 106 kb) [file 12879_2016_1553_MOESM2_ESM.doc]
